# Vasculopathy Augments Cardiovascular Risk in Community-Dwelling Elderly with Left Ventricular Hypertrophy

**DOI:** 10.3390/jpm14060558

**Published:** 2024-05-23

**Authors:** Rusitanmujiang Maimaitiaili, Song Zhao, Jiadela Teliewubai, Shikai Yu, Weilun Meng, Yifan Zhao, Yawei Xu, Yi Zhang

**Affiliations:** Department of Cardiology, Shanghai Tenth People’s Hospital, Tongji University School of Medicine, 301 Middle Yanchang Road, Shanghai 200072, China

**Keywords:** vasculopathies, left ventricular hypertrophy, cardiovascular risk, elderly

## Abstract

This study aimed to investigate the impact of various vasculopathies alongside left ventricular hypertrophy (LVH) on cardiovascular risk in the elderly. This prospective cohort study included 3339 older adults from the Northern Shanghai Study, classified into four mutually exclusive left ventricular (LV) geometry groups based on echocardiographic data: normal geometry, concentric remodeling, eccentric hypertrophy, and concentric hypertrophy. Vasculopathy was categorized into three primary types: arteriosclerosis, atherosclerosis, and renal senescence. Major adverse cardiovascular events (MACEs) were defined as non-fatal acute myocardial infarction, non-fatal stroke, and cardiovascular deaths according to ICD-10 codes. Over a median follow-up period of 5.7 years, 221 incident cases of MACEs were identified. Concentric hypertrophy exhibited the highest prevalence of hypertension, the most significant increase in vascular stiffness, and the highest rate of MACEs. The adjusted Cox regression analysis showed that eccentric hypertrophy is associated with an increased risk of MACEs (HR: 1.638 [95% CI: 1.151–2.331], *p* = 0.006), while concentric hypertrophy shows an even higher risk (HR: 1.751 [95% CI: 1.127–2.721], *p* = 0.013). Conversely, concentric remodeling was not significantly associated with an increased risk of MACEs. Renal senescence presents a moderate but significant risk for MACEs, with an HR of 1.361 (95% CI: 1.019–1.819; *p* = 0.037) when adjusted for LVH. The Kaplan–Meier analysis showed that patients with LVH and multiple vasculopathies experience the most significant decrease in survival probability (log-rank *p* < 0.001). The subgroup analysis revealed that LVH significantly raises the risk of MACEs, especially in older males with hypertension, diabetes, or vasculopathy. This study reinforces the importance of LVH as a predictor of adverse cardiovascular outcomes and underscores the compounded risk associated with the presence of multiple vasculopathies. Additionally, it highlights renal senescence as a distinct and independent risk factor for MACEs, separate from LVH.

## 1. Introduction

Alterations in left ventricular (LV) geometry are increasingly observed within aging populations, signifying a range of cardiac structural adaptations to both physiological and pathophysiological stimuli [1,2]. Among these structural modifications, left ventricular hypertrophy (LVH) emerges as a particularly salient indicator of cardiac morbidity, acting as a significant independent prognostic marker for cardiovascular (CV) events in geriatric cohorts [3].

Arteriosclerosis, characterized by the hardening of arterial walls, is widely recognized as a predominant risk factor for adverse cardiovascular outcomes [4]. This condition not only serves as a principal contributor to arterial hypertension in the elderly but also precipitates a spectrum of detrimental effects on the cardiovascular and cerebrovascular systems [5,6]. Atherosclerosis, another form of arterial damage, involves the accumulation of lipids, cholesterol, and cellular debris within the arterial walls. This accumulation forms plaques that narrow and stiffen arteries, also impeding blood flow and increasing the risk of adverse cardiovascular events [7,8]. Renal senescence describes the progressive decline in kidney function and structural integrity, marked by a decreased estimated glomerular filtration rate (eGFR) and alterations in renal blood flow, also notably elevating cardiovascular risk [9,10].

LVH is known to precede cardiovascular problems, yet how much vasculopathies such as arteriosclerosis, atherosclerosis, and renal senescence intensify this risk needs deeper exploration. The significance of arteriosclerosis and atherosclerosis among individuals with LVH has been increasingly recognized, underscoring the need for a detailed study of their interactions [11,12,13]. Renal senescence, another vasculopathy often linked with higher cardiovascular risk, is commonly observed alongside LVH [14,15,16]. While previous research has connected LVH with kidney-related issues like chronic kidney disease and microalbuminuria [17,18], offering valuable insights, the overall increase in cardiovascular risk posed by the combination of LVH with various vasculopathies remains unclear. Considering the prevalence of these coexisting conditions in the elderly, it is imperative to thoroughly investigate these complex relationships to enhance our understanding and approach to managing cardiovascular risk in this demographic.

Thus, this study aims to understand how vasculopathy alongside LVH impacts cardiovascular risk in the elderly. We investigate how different vascular diseases affect this risk, proposing that a greater variety and severity of these conditions could lead to higher health risks. Our goal is to improve how we identify and manage these risks, potentially leading to better, more personalized care for the elderly.

## 2. Materials and Methods

### 2.1. Participants

All participants in this study were recruited from the Northern Shanghai Study (ClinicalTrials.gov Identifier: NCT02368938), a population-based study focused on elderly living in the community. The study design, inclusion, and exclusion criteria were described previously [19]. The inclusion criteria were as follows: (1) age 65 years or more, (2) local residents from urban communities, and (3) willing to sign the informed consent and available for long-term follow-up. The exclusion criteria were (1) with serious heart disease (NYHA IV) or end-stage renal disease (CKD ≥ 4 stage), (2) suffered from cancer or life expectancy less than 5 years, and (3) stroke history within 3 months.

A total of 3363 patients were enrolled between June 2014 and May 2019. Twenty-four individuals were excluded due to missing crucial data, resulting in a final sample size of 3339 for this study.

### 2.2. Demographic, Clinical, and Biological Data

The study gathered demographic data through participants’ identification. The standardized structured questionnaire was used to obtain past medical history, which included histories of hypertension, diabetes mellitus, previous coronary heart disease (CHD), smoking, and alcohol history. Body height and weight were measured using regular methods, and body mass index (BMI) was calculated; obese was defined as BMI ≥ 28 kg/m^2^ [20]. Fasting blood and urine samples were collected on the morning of the check-up. Hypertension was identified when individuals exhibited a blood pressure reading greater than 140/90 mmHg, measured under standardized conditions. To minimize the impact of white coat hypertension, blood pressure measurements were taken after a 5-min rest period, and the average of the second and third readings was used for diagnosis. Additionally, participants were asked about their blood pressure history and any ongoing antihypertensive treatment to further validate the diagnosis [21]. The biological samples were examined at the Laboratory Medicine Department of Shanghai Tenth People’s Hospital, which adheres to stringent quality control standards and follows validated protocols to ensure the accuracy and reliability of all test results, ensuring compliance with both local and international laboratory practices. The diagnosis of diabetes mellitus was made for participants whose fasting blood glucose levels were 7.0 mmol/L or higher, or who were on insulin or other glucose-lowering drugs.

### 2.3. Echocardiography and Definition of LV Geometry

Trained cardiologists measured ultrasound parameters using the Mylab 30 Gold cardiovascular system (ESAOTE SpA, Genoa, Italy). To determine LV diameters, M-mode and two-dimensional echocardiography were conducted with a 3.5-MHz probe, following the American Society of Echocardiography’s guidelines [22]. LV end-diastolic diameter (LVEDd), interventricular septal diameter (IVSd), and posterior wall thickness diameter (PWTd) were, respectively, measured from the parasternal view at end-diastole. LV ejection fraction (LVEF) and LV volume were measured by M-mode echocardiography in long axis view, using the adjusted Teichholz formula [23].

LV mass was calculated based on standard methods currently recommended [22]: $LV\;mass=0.8\times \left\{\left.1.04\times \left[\left.{\left(IVSd+LVEDd+\left.PWTd\right)\right.}^{3}-{LVEDd}^{3}\right]\right.\right\}+0.6\right.$. LV mass was then indexed to body surface area (BSA). Relative wall thickness (RWT) was calculated as 2 × PWTd/LVEDd [22].

LV geometry was classified into four categories based on LVMI and RWT measurements (Table 1). RWT values between 0.32 and 0.42, and LVMI values of ≤115 g/m^2^ for males and ≤95 g/m^2^ for females indicated normal geometry. Values exceeding these thresholds, such as LVMI > 115 g/m^2^ for males or > 95 g/m^2^ for females and RWT < 0.32 or RWT > 0.42, were considered indicative of altered LV dimensions. LV geometry was further defined as normal (characterized by normal LVMI and RWT), concentric remodeling (characterized by normal LVMI and RWT above 0.42), eccentric hypertrophy (characterized by increased LVMI and RWT below 0.42), and concentric hypertrophy (characterized by increased LVMI and RWT above 0.42) [22].

### 2.4. Definition of Vasculopathy

The carotid–femoral pulse wave velocity (cfPWV) was measured through applanation tonometry using SphygmoCor (AtCor Medical, Sydney, Australia) [24] by a trained researcher. ABI (ankle-brachial index) is determined using the VP-1000 system (Omron, Japan) [25]. The carotid intima-media thickness (cIMT) and presence of carotid plaques were assessed using a 7.5 MHz ultrasound probe on a Mylab 30 Gold cardiovascular system (ESAOTE SpA, Genoa, Italy) [26]. The mean value was computed from three separate readings for accuracy. In addition, the examination included a determination of whether carotid plaques were present or absent. Vasculopathy was then classified into three primary types: arteriosclerosis, characterized by a cfPWV equal to or exceeding 10 m/s [5]; atherosclerosis was identified by the presence of carotid plaque, an ABI of less than 0.9—using the lower measurement between the left or right ABI—or a cIMT equal to or greater than 0.9 mm [27,28]; and renal senescence, determined by laboratory results indicating an estimated glomerular filtration rate (eGFR) of 60 mL/min/1.73 m^2^ or less, or a urinary albumin-to-creatinine ratio (UACR) exceeding 30 mg/g [29].

### 2.5. Clinical Outcomes

Participants were followed for MACEs up to 8.2 years from baseline examinations. MACE was defined as the first occurrence of any of the following events: non-fatal acute myocardial infarction (AMI), non-fatal stroke, and CV deaths according to ICD10 code.

### 2.6. Statistical Analysis

Data are presented as frequencies and proportions for categorical variables, and medians with interquartile ranges (IQRs) for continuous variables due to non-normal distribution confirmed by Shapiro–Wilk tests. For analyzing trends across ordered groups defined by LV geometry, the Jonckheere–Terpstra test was employed to assess if there is a significant trend in the median values. Group differences were evaluated using Kruskal–Wallis tests for continuous variables and Chi-square tests for categorical variables. Post hoc analyses were performed using Dunn’s test with Bonferroni correction for multiple comparisons where necessary. Associations between various forms of vasculopathy (arteriosclerosis, atherosclerosis, and renal senescence) and MACEs were examined using Cox proportional hazards models, calculating hazard ratios (HRs) and 95% confidence intervals (CIs), while adjusting for potential confounders. Interaction terms were also explored to assess potential synergistic effects on cardiovascular risk. Sensitivity analyses were conducted to validate the robustness of the findings, focusing on age and severity of LVH and vasculopathies. Two-sided *p*-values < 0.05 were considered significant. Statistical tests were performed with R version 4.2.2 (R Foundation for Statistical Computing, Vienna, Austria) and SPSS version 27.0 (IBM Corp., Armonk, NY, USA).

## 3. Results

### 3.1. Baseline Characteristics

Table 2 demonstrates a trend across different left ventricular geometry groups. Age increased from a median of 69 years in the normal geometry group to 71 years in the concentric hypertrophy group (*p* for trend <0.001). BMI also showed a slight upward trend from 24 kg/m^2^ in the normal geometry group to 25 kg/m^2^ in the eccentric hypertrophy group (*p* for trend <0.001). Smoking history decreased significantly from 27.7% in the normal group to 13.2% in the concentric hypertrophy group (*p* for trend <0.001), and systolic blood pressure rose from 134 mm Hg to 140 mm Hg across the groups (*p* for trend <0.001). Laboratory results showed a minimal upward trend in cholesterol and stable uric acid levels. Notably, LVMI increased from 76 g/m^2^ in the normal group to 120 g/m^2^ in the eccentric hypertrophy group (*p* for trend <0.001), while LVEF slightly decreased (*p* for trend <0.001). Prevalence of hypertension and MACEs was highest in the concentric hypertrophy group, 78.8% and 11.2%, respectively (*p* for trend <0.001), underscoring the link between more severe LV geometry and higher cardiovascular risks.

### 3.2. Prevalence of Vasculopathy Stratified by LV Geometry

**Figure 1:**
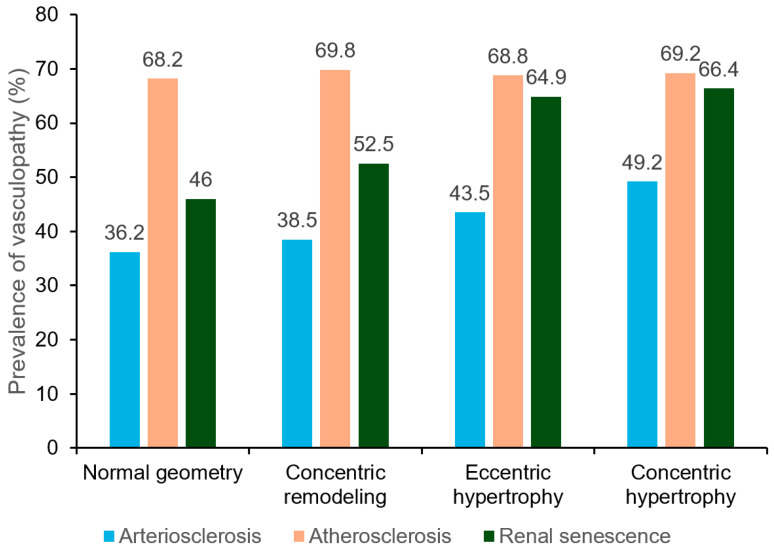
Prevalence of vasculopathy stratified by left ventricular geometry.

The bar graph delineates the prevalence of vasculopathy categorized into arteriosclerosis, atherosclerosis, and renal senescence across four types of left ventricular geometry (Figure 1). In the normal geometry group, arteriosclerosis prevalence is noted at 36.2%, while atherosclerosis and renal senescence are at 68.2% and 46%, respectively. The concentric remodeling group shows a similar trend with arteriosclerosis at 38.5%, but a higher prevalence of atherosclerosis at 69.8% and renal senescence at 52.5%. For eccentric hypertrophy, arteriosclerosis prevalence dips slightly to 43.5%, atherosclerosis remains consistent at 68.8%, and renal senescence increases to 64.9%. Lastly, the concentric hypertrophy group maintains a prevalence of 49.2% for arteriosclerosis, shows a slight increase in atherosclerosis to 69.2%, and exhibits the highest prevalence of renal senescence at 66.4%. The analysis revealed distinct trends in the prevalence of vasculopathies with increasing severity of cardiac remodeling: arteriosclerosis and renal senescence both showed a statistically significant increasing trend (*p* < 0.001 for both), whereas the trend for atherosclerosis was not statistically significant (*p* = 0.723).

### 3.3. Association of Left Ventricular Geometry with MACEs

Over a median follow-up period of 5.7 years, 224 incident cases of MACEs were identified. Multivariate Cox regression models were used to evaluate the occurrence of MACEs across different left ventricular geometries compared to the reference group (Table 3). Across four adjusted models, the data indicate an increased risk of MACEs in both eccentric and concentric hypertrophy groups. In the most fully adjusted model (Model 4), the eccentric hypertrophy group showed an HR of 1.638 (95% CI: 1.151–2.331; *p* = 0.006), and the concentric hypertrophy group showed an even higher HR of 1.751 (95% CI: 1.127–2.721; *p* = 0.013). Concentric remodeling did not show a statistically significant association with MACEs in any model, with the HR in Model 4 being 1.167 (95% CI: 0.825–1.651; *p* = 0.384). This suggests that while concentric remodeling may not significantly increase the risk of MACEs, both eccentric and concentric hypertrophy are associated with a significant escalation in risk, with concentric hypertrophy presenting the highest hazard among the groups.

### 3.4. Cardiovascular Risk of LVH along with Vasculopathies

A Cox regression model was used to evaluate the association between LVH and different vasculopathy conditions with the risk of MACEs (Table 4). LVH is associated with a significant increase in the risk of MACEs, as indicated by an HR of 1.585 (95% CI: 1.186–2.119; *p* = 0.002). In contrast, arteriosclerosis and atherosclerosis, with HRs of 1.140 (95% CI: 0.857–1.515; *p* = 0.368) and 1.187 (95% CI: 0.859–1.639; *p* = 0.299), respectively, do not show a statistically significant association with MACEs. However, renal senescence presents a moderate but significant risk for MACEs with an HR of 1.361 (95% CI: 1.019–1.819; *p* = 0.037). These findings suggest that while LVH and renal senescence are significant predictors of MACEs, the presence of arteriosclerosis or atherosclerosis alone does not confer a statistically significant risk for these events.

### 3.5. Cardiovascular Risks Associated with LVH in the Presence of Concurrent Vasculopathies

**Figure 2:**
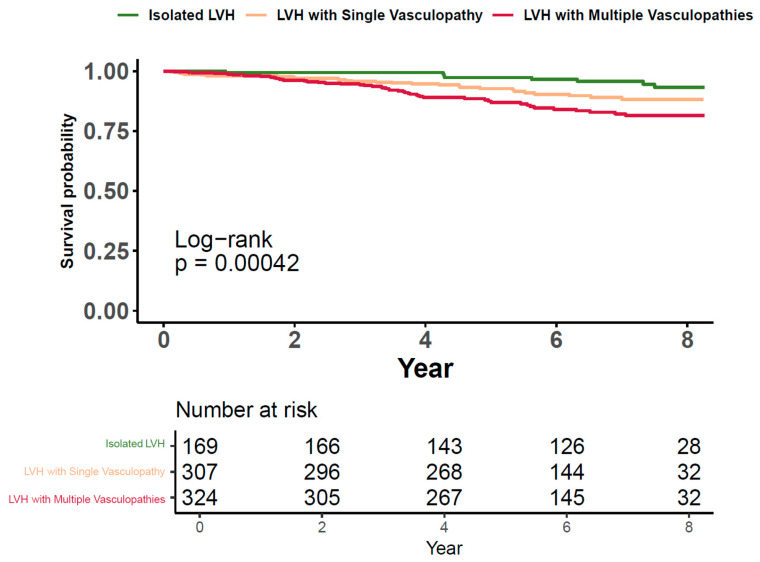
Kaplan–Meier analysis for LVH Patients with vasculopathies. **Abbreviations:** LVH, left ventricular hypertrophy.

The Kaplan–Meier curve depicts the survival probability for patients categorized by the presence of LVH and concurrent vasculopathies (Figure 2). Three groups are compared: those with isolated LVH, LVH with a single vasculopathy, and LVH with multiple vasculopathies. Patients with isolated LVH exhibit the highest survival probability, maintaining close to baseline survival over the eight years. Those with LVH and a single vasculopathy demonstrate a modest reduction in survival probability, while patients with LVH and multiple vasculopathies show a more pronounced decline. The log-rank test confirms a statistically significant difference in survival distributions among the three groups (*p* = 0.00042), indicating that the addition of vasculopathies to LVH is associated with decreased survival. Data entries lacking information on vasculopathy conditions or MACEs were omitted from this analysis.

### 3.6. Impact of LVH on MACEs across Various Subgroups

**Figure 3:**
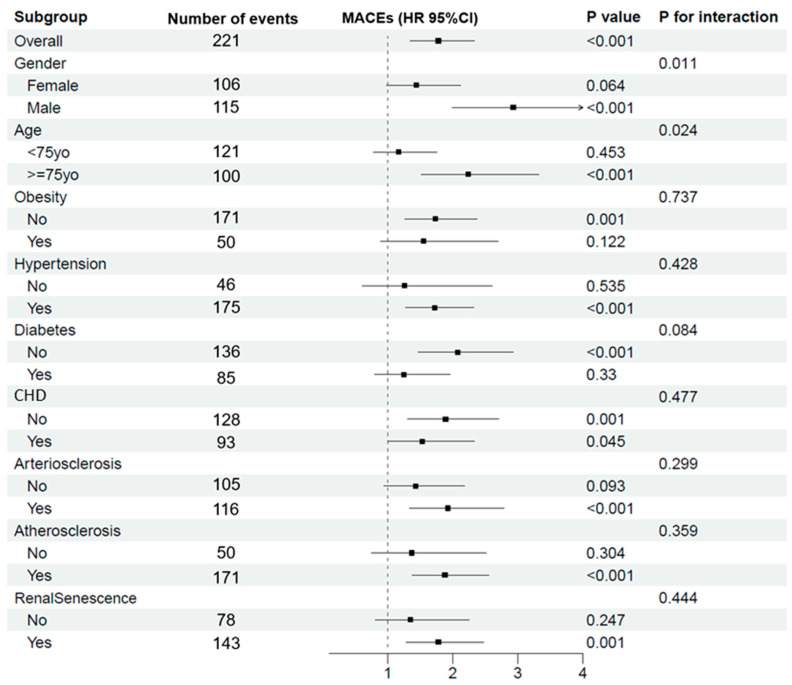
Subgroup analysis for LVH on the occurrence of MACEs. **Abbreviations:** LVH, left ventricular hypertrophy; MACEs, major adverse cardiovascular events; CHD, coronary heart disease; HR, hazard ratio; CI, confidence interval.

Figure 3 shows the influence of LVH on the occurrence of major MACEs within different subgroups. Overall, LVH poses a substantial risk (*p* < 0.001). By gender, males show a higher propensity for MACEs (*p* < 0.001), with a noted interaction effect (*p* = 0.011). The age-related analysis indicates that patients 75 years and older are at an elevated risk (*p* < 0.001), and this risk is influenced by very old age (*p* for interaction = 0.024). For other health conditions, LVH patients with hypertension and diabetes exhibit a significantly increased risk of MACEs (*p* < 0.001), whereas obesity does not significantly contribute to this risk (*p* = 0.122). Both the presence and absence of CHD increase the risk of MACEs in patients with LVH, with a marginally higher risk observed in those without CHD. This may be related to the effect of medications or interventions for CHD that could lower the risk of MACEs or other underlying health factors not accounted for in the surface data. Finally, the presence of vasculopathy significantly increased the risk for MACEs in participants with LVH.

## 4. Discussion

In this cohort of older, community-dwelling individuals, the study established that LVH still remains an independent and strong predictor of MACEs. The presence of multiple vasculopathy conditions incrementally increased the risk of MACEs in patients with LVH, indicating a compounded effect of these conditions in the elderly population. The study also brought to light that renal senescence has a distinct association with the risk of MACEs, separate from the influence of LVH. This underscores the importance of considering both cardiac and renal health in cardiovascular risk stratification in elderly.

Consistent with prior research, our findings once again emphasized the prognostic significance of LVH, which has been long established as a cardinal marker of increased cardiovascular morbidity and mortality [1,2,3]. While our findings corroborate the well-documented prognostic significance of LVH in predicting cardiovascular outcomes, an intriguing aspect of our analysis was the absence of a significant risk increase in the subgroup exhibiting concentric remodeling. This subgroup, traditionally characterized by an increased relative wall thickness without an increase in left ventricular mass, did not demonstrate the heightened risk for MACEs that might be expected. This could suggest that the structural adaptations associated with concentric remodeling do not confer the same degree of risk as other forms of LVH in this cohort. However, there are other studies that presented similar result [30,31].

The incremental increase in the risk of MACEs with the presence of multiple vasculopathy conditions highlights the compounded nature of cardiovascular risk in elderly patients who suffer from LVH. Each additional vasculopathy does not merely add to the risk in a linear fashion but may amplify it, suggesting a possible synergistic interaction between these conditions. This is particularly relevant for the elderly population, where the cumulative effect of several coexisting vasculopathies—such as atherosclerosis, arteriosclerosis, and renal senescence—can lead to a significantly higher risk of adverse cardiovascular outcomes [4,5,6,7,8,9,10]. The biological underpinnings for this compounded effect may involve shared pathogenic pathways. For instance, LVH often results from chronic pressure overload, which can be exacerbated by the arterial stiffness seen in arteriosclerosis and atherosclerosis [15,16,32]. Renal senescence might further compound these effects through the interplay of hypertension, dysregulated volume control, and hormonal disturbances affecting cardiovascular health [33,34,35]. Clinically, this finding highlights the necessity for a comprehensive approach to managing elderly patients with LVH. It points to the importance of screening for and addressing multiple vascular pathologies, not just in isolation but as a collective suite of risk factors that may precipitate MACEs. The data thus advocate for a multidisciplinary management strategy, possibly involving cardiologists, nephrologists, and primary care providers to mitigate this elevated risk.

Our study’s findings on renal senescence elucidate a nuanced aspect of cardiovascular risk stratification, notably in the context of aging. The independent association between renal senescence and the risk of MACEs, irrespective of LVH, accentuates the critical role of renal health in cardiovascular disease (CVD) prognostication. Renal senescence often goes together with a decline in the glomerular filtration rate and an increase in fibrotic tissue, both of which can impair the kidneys’ ability to modulate blood pressure and volume status effectively [34]. Additionally, aging kidneys may release more renin, contributing to the renin–angiotensin–aldosterone system (RAAS)’s overactivation, leading to further endothelial dysfunction and arterial stiffness—factors known to contribute to CVD [36]. The clinical implication of this finding is substantial. It suggests that, in elderly patients, especially those with evidence of LVH, a comprehensive assessment that includes evaluating renal function may be warranted. Identifying renal senescence early could provide a window of opportunity for intervention before the development of overt cardiovascular disease or before the acceleration of existing CVD.

In this study, it is crucial to consider the role of antihypertensive treatment in managing the progression of LVH [37]. As observed in our cohort, participants receiving robust antihypertensive treatment showed varied progression rates of LVH, highlighting the importance of effective blood pressure management. Furthermore, inadequate control of hypertension, a known contributor to LVH [38], was evident in a subset of our participants. This underscores the need for stringent blood pressure monitoring and management to mitigate the risk and severity of LVH. Additionally, factors such as age, genetic predispositions, and lifestyle choices also play critical roles in the development of LVH and should be considered when devising treatment and management strategies [39,40].

The novelty of our study lies in its elucidation of the relationship between LVH and multiple vasculopathies, wherein a higher number of vasculopathy conditions correlates with an increased risk of MACEs. This association suggests a possible synergistic effect of concurrent vascular conditions, amplifying the risk posed by LVH alone.

Our study stands out for its extensive analysis of a large group of elderly, community-dwelling individuals and its novel focus on how multiple vasculopathies, including arteriosclerosis, atherosclerosis, and renal senescence, interact with LVH to impact cardiovascular risk. By exploring these complex interactions and their role in MACEs, we highlight the critical importance of considering both cardiac and renal health in assessing cardiovascular risk in the elderly. The thoroughness of our methodology—from detailed clinical measurements to careful statistical analysis—ensures the reliability of our findings. These insights not only confirm the significant role of LVH as a predictor of cardiovascular outcomes but also pave the way for future research into more integrated approaches for managing cardiovascular health, emphasizing a comprehensive treatment strategy that includes managing blood pressure, heart function, and kidney health.

Acknowledging the limitations of our study, it is important to note that its observational nature restricts our ability to infer causality between LVH, vasculopathies, and MACEs. Additionally, the specific cohort of elderly, community-dwelling individuals may not fully represent the broader elderly population. Measurement constraints and the potential for unaccounted confounding variables further call for a cautious interpretation of the findings. Despite these considerations, the study contributes valuable insights into cardiovascular risk stratification, underscoring the importance of further research in this area.

## 5. Conclusions

This study reinforces the importance of LVH as a predictor of adverse cardiovascular outcomes and underscores the compounded risk associated with the presence of multiple vasculopathies. Additionally, this study highlights renal senescence as a distinct and independent risk factor for MACEs, separate from LVH.

## Figures and Tables

**Table 1:** Definition of left ventricular geometry.

Left Ventricular Geometry	LVMI (g/m^2^)	RWT
**Normal**	<115 for male <95 for female	≤0.42
**Concentric remodeling**	<115 for male <95 for female	>0.42
**Eccentric hypertrophy**	≥115 for male ≥95 for female	≤0.42
**Concentric hypertrophy**	≥115 for male ≥95 for female	>0.42

**Abbreviations:** LVMI, left ventricular mass index; RWT, relative wall thickness.

**Table 2:** Baseline characteristics.

		Normal Geometry (n = 1761)	Concentric Remodeling (n = 775)	Eccentric Hypertrophy (n = 552)	Concentric Hypertrophy (n = 251)	*p* for Trend
**Basic characteristics**						
	Age (years)	69 (66–73)	69 (67–74)	70 (67–76)	71 (67–78)	<0.001
	Male (n, %)	859 (48.8)	378 (48.8)	154 (27.9)	62 (24.8)	<0.001
	BMI (kg/m^2^)	24 (22–26)	24 (21–26)	25 (22–27)	24 (22–27)	<0.001
	Alcohol history (n, %)	322 (18.3)	135 (17.4)	70 (12.7)	30 (12.0)	<0.001
	Smoking history (n, %)	488 (27.7)	210 (27.1)	92 (16.7)	33 (13.2)	<0.001
**Blood pressure**						
	Systolic blood pressure (mm Hg)	134 (122–145)	131 (120–145)	139 (127–150)	140 (126–151)	<0.001
	Diastolic blood pressure (mm Hg)	80 (72–86)	80 (71–84)	80 (71–85)	80 (73–85)	0.275
**Laboratory blood results**						
	FBG (mmol/L)	5.2 (4.8–5.9)	5.3 (4.8–6.1)	5.3 (4.8–6.1)	5.3 (4.8–6.5)	0.026
	Total cholesterol (mmol/L)	5.1 (4.4–5.7)	5.0 (4.4–5.7)	5.1 (4.5–5.7)	5.3 (4.5–5.9)	0.193
	Triglyceride (mmol/L)	1.4 (1.0–1.9)	1.4 (1.0–1.9)	1.4 (1.1–1.9)	1.4 (1.1–2.0)	0.126
	Uric acid (mmol/L)	325 (274–379)	322 (277–380)	321 (276–374)	323 (264–382)	0.940
**Left ventricular parameters**						
	LVMI (g/m^2^)	76 (64–88)	77 (64–88)	120 (105–133)	119 (107–133)	<0.001
	RWT	0.35 (0.32–0.38)	0.47 (0.44–0.51)	0.36 (0.32–0.39)	0.47 (0.44–0.52)	<0.001
	LVEF (%)	59 (51–68)	57 (50–66)	56 (50–63)	58 (51–64)	<0.001
**Pre-existing diseases**						
	Hypertension (n, %)	1132 (64.2)	485 (62.6)	432 (78.3)	197 (78.8)	<0.001
	Diabetes (n, %)	399 (22.6)	199 (25.7)	160 (29.0)	80 (32.0)	<0.001
	CHD (n, %)	519 (29.5)	245 (31.6)	216 (39.3)	91 (36.4)	<0.001
**Vasculopathy parameters**						
	UACR (mg/g)	25 (12–51)	30 (16–57)	42 (21–76)	38 (23–79)	<0.001
	cfPWV (m/s)	8.8 (7.6–10.2)	8.9 (7.9–10.6)	9.5 (8.1–10.9)	9.7 (8.3–11.2)	<0.001
	R-ABI	1.09 (1.02–1.15)	1.08 (0.99–1.14)	1.08 (1.01–1.15)	1.06 (0.98–1.13)	<0.001
	L-ABI	1.08 (1.01–1.14)	1.06 (0.97–1.13)	1.08 (0.99–1.14)	1.03 (0.96–1.10)	<0.001
	eGFR (mL/min/1.73/m^2^)	85 (75–96)	84 (74–96)	84 (73–95)	83 (71–95)	0.003
	cIMT (mm)	0.64 (0.55–0.77)	0.64 (0.54–0.76)	0.61 (0.52–0.74)	0.64 (0.53–0.74)	0.008
	Carotid plaque (n, %)	1169 (66.3)	521 (66.2)	361 (65.4)	162 (64.8)	0.614
**Events**						
	MACEs	87 (4.9)	52 (6.7)	54 (9.8)	28 (11.2)	<0.001

**Abbreviations:** BMI, body mass index; FBG, fasting blood glucose; LVMI, left ventricular mass index; RWT, relative wall thickness; LVEF, left ventricular ejection fraction; CHD, coronary heart disease; UACR, urine albumin-to-creatinine ratio; cfPWV, carotid-femoral pulse wave velocity; R-ABI, right ankle-brachial index; L-ABI, left ankle-brachial index; eGFR, estimated glomerular filtration rate; cIMT, carotid intima-media thickness; MACEs, major adverse cardiovascular events.

**Table 3:** Association of left ventricular geometry with MACEs.

	Normal Geometry	Concentric Remodeling		Eccentric Hypertrophy		Concentric Hypertrophy	
MACEs	ref	HR (95% CI)	*p*	HR (95% CI)	*p*	HR (95% CI)	*p*
Model 1	1.000	1.256 (0.891–1.772)	0.193	1.866 (1.329–2.622)	<0.001	2.042 (1.333–3.127)	0.001
Model 2	1.000	1.183 (0.837–1.673)	0.342	1.776 (1.249–2.525)	0.001	1.868 (1.203–2.899)	0.005
Model 3	1.000	1.176 (0.831–1.663)	0.361	1.675 (1.178–2.381)	0.004	1.783 (1.148–2.771)	0.010
Model 4	1.000	1.167 (0.825–1.651)	0.384	1.638 (1.151–2.331)	0.006	1.751 (1.127–2.721)	0.013

**Model 1:** univariate. **Model 2:** adjusted for age and gender. BMI **Model 3:** adjusted for age, gender, BMI, hypertension, diabetes, CHD, and smoking history. **Model 4:** adjusted for age, gender, BMI, hypertension, diabetes, CHD, smoking history, arteriosclerosis, atherosclerosis, and renal senescence. **Abbreviations:** BMI, body mass index; CHD, coronary heart disease; MACEs, major adverse cardiovascular events; HR, hazard ratio; CI, confidence interval.

**Table 4:** Association of left ventricular hypertrophy and vasculopathy conditions with MACEs.

	HR	95% CI	*p*
LVH	1.585	1.186–2.119	0.002
Arteriosclerosis	1.140	0.857–1.515	0.368
Atherosclerosis	1.187	0.859–1.639	0.299
Renal senescence	1.361	1.019–1.819	0.037

Adjusted for age, gender, BMI, hypertension, diabetes, CHD, and smoking history. **Abbreviations**: BMI, body mass index; CHD, coronary heart disease; MACEs, major adverse cardiovascular events. LVH, left ventricular hypertrophy; HR, hazard ratio; CI, confidence interval.

## Data Availability

The participant data of this study/trial are not to be shared due to some limitations, and no other study-related document will be made available.
